# The Free Zinc Concentration in the Synaptic Cleft of Artificial Glycinergic Synapses Rises to At least 1 μM

**DOI:** 10.3389/fnmol.2016.00088

**Published:** 2016-09-22

**Authors:** Yan Zhang, Angelo Keramidas, Joseph W. Lynch

**Affiliations:** ^1^Queensland Brain Institute, The University of Queensland, BrisbaneQLD, Australia; ^2^School of Biomedical Sciences, The University of Queensland, BrisbaneQLD, Australia

**Keywords:** glycine receptor, chloride channel, zinc chelator, inhibitory synapse, tricine, ZX1

## Abstract

Zn^2+^ is concentrated into presynaptic vesicles at many central synapses and is released into the synaptic cleft by nerve terminal stimulation. There is strong evidence that synaptically released Zn^2+^ modulates glutamatergic neurotransmission, although there is debate concerning the peak concentration it reaches in the synaptic cleft. Glycine receptors (GlyRs), which mediate inhibitory neurotransmission in the spinal cord and brainstem, are potentiated by low nanomolar Zn^2+^ and inhibited by micromolar Zn^2+^. Mutations that selectively ablate Zn^2+^ potentiation result in hyperekplexia phenotypes suggesting that Zn^2+^ is a physiological regulator of glycinergic neurotransmission. There is, however, little evidence that Zn^2+^ is stored presynaptically at glycinergic terminals and an alternate possibility is that GlyRs are modulated by constitutively bound Zn^2+^. We sought to estimate the peak Zn^2+^ concentration in the glycinergic synaptic cleft as a means of evaluating whether it is likely to be synaptically released. We employed ‘artificial’ synapses because they permit the insertion of engineered α1β GlyRs with defined Zn^2+^ sensitivities into synapses. By comparing the effect of Zn^2+^ chelation on glycinergic IPSCs with the effects of defined Zn^2+^ and glycine concentrations applied rapidly to the same recombinant GlyRs in outside-out patches, we inferred that synaptic Zn^2+^ rises to at least 1 μM following a single presynaptic stimulation. Moreover, using the fast, high-affinity chelator, ZX1, we found no evidence for tonic Zn^2+^ bound constitutively to high affinity GlyR binding sites. We conclude that diffusible Zn^2+^ reaches 1 μM or higher and is therefore likely to be phasically released in artificial glycinergic synapses.

## Introduction

The free ionic form of zinc (Zn^2+^) is present in the extracellular CNS solution at low nM concentrations (Frederickson et al., 2006). We define this as ‘tonic’ Zn^2+^. It is also concentrated into presynaptic vesicles at many brain glutamatergic synapses (Frederickson, 1989) and is released into the synaptic cleft by nerve terminal stimulation (Assaf and Chung, 1984; Howell et al., 1984). The GluN2A-containing NMDA receptor (NMDAR) cation channels that populate these synapses are inhibited by Zn^2+^ at low nM concentrations (Paoletti et al., 1997). Recent studies have found evidence for the transient modulation of synaptic NMDARs by phasically released Zn^2+^ (Vergnano et al., 2014; Anderson et al., 2015) and for the constitutive modulation of extrasynaptic NMDARs by tonic Zn^2+^ (Anderson et al., 2015). A major unresolved question concerns the concentration to which Zn^2+^ rises during synaptic activation, with estimates spanning four orders of magnitude from 10 nM (Kay, 2003) to >100 μM (Vogt et al., 2000). It is important to resolve this because many neuronal membrane proteins are modulated by Zn^2+^ at high concentrations (Marger et al., 2014), and a knowledge of the peak Zn^2+^ concentration would permit the identification of potential physiological targets of synaptically released Zn^2+^.

It is also unclear whether tonic or synaptically released Zn^2+^ modulates neurotransmission at other synapses. Glycine receptor (GlyR) chloride channels, which mediate inhibitory neurotransmission in the spinal cord and brainstem (Lynch, 2004), are a likely candidate for Zn^2+^ modulation for several reasons. First, the major synaptic α1β GlyR isoform is extremely sensitive to Zn^2+^, with low (10–1000 nM) Zn^2+^ concentrations potentiating EC_50_ glycine-gated currents and higher Zn^2+^ concentrations (3–300 μM) eliciting a dose-dependent inhibition (Bloomenthal et al., 1994; Laube et al., 1995; Miller et al., 2005a,b). Second, selectively ablating the sensitivity of α1 GlyRs to Zn^2+^ potentiation led to impaired glycinergic inhibition and a hyperekplexia-type phenotype in α1^D80A^ GlyR knock-in mice (Hirzel et al., 2006). Third, by eliminating free Zn^2+^ from the synaptic cleft via Zn^2+^ buffers and chelators, it has been shown that glycinergic inhibitory post-synaptic currents (IPSCs) are positively modulated by either tonic or synaptically released Zn^2+^ (Suwa et al., 2001; Hirzel et al., 2006; Perez-Rosello et al., 2015). Fourth, there is strong evidence for the co-localization of Zn^2+^ and glycine at presynaptic terminals in the spinal cord of the lamprey (Birinyi et al., 2001) although the evidence for their co-localization at glycinergic presynaptic terminals in the mammalian spinal cord is less clear cut (Wang et al., 2001). However, to date there has been no investigation into the possibility that Zn^2+^ may be phasically released at glycinergic synapses.

Since glycine reaches a saturating concentration in the cleft during synaptic stimulation (Legendre, 1998; Beato, 2008), potentiating concentrations of Zn^2+^ cannot further increase the IPSC magnitude, and as a result Zn^2+^ potentiation is manifest as a slowing in the IPSC decay rate (Suwa et al., 2001; Laube, 2002; Hirzel et al., 2006; Eto et al., 2007). However, miniature IPSCs, which are mediated by the stochastic release of single glycine vesicles and thus involve sub-saturating glycine concentrations, are increased in magnitude by potentiating concentrations of Zn^2+^ (Suwa et al., 2001; Hirzel et al., 2006; Perez-Rosello et al., 2015).

We have recently developed an ‘artificial’ synapse system whereby glycinergic synapses are induced to form between presynaptic terminals of cultured spinal glycinergic interneurons and HEK293 cells expressing recombinant heteromeric GlyRs (Dixon et al., 2015; Zhang et al., 2015). This system has two main advantages over neuronal synapses. First, it allows control over the subunit composition of post-synaptic GlyRs. Second, the electrotonically compact shape of HEK293 cells avoids problems associated with dendritic filtering, allowing IPSC waveforms to be resolved with high fidelity. In the present study, we engineered the Zn^2+^ sensitivities of recombinant GlyRs so that they could act as reporters of synaptic Zn^2+^ over different concentration ranges. By comparing the effect of Zn^2+^ chelation on IPSCs with the effects of buffered Zn^2+^ plus glycine concentrations applied rapidly to the same recombinant GlyRs to simulate synaptic activation, we sought to provide an estimate of the concentration to which Zn^2+^ rises in response to a single presynaptic stimulation.

## Materials and Methods

### Molecular Biology and HEK293 Cell Transfection

We employed plasmid DNAs encoding the human α1 (pCIS), rat α3L (pcDNA3.1) and human β (pcDNA3.1) GlyR subunits, plus mouse neuroligin 2A (pNice) and rat gephyrin P1 (pCIS). Empty pEGFP plasmid was also transfected as an expression marker. Site-directed mutagenesis was performed using the QuikChange mutagenesis kit (Agilent Technologies) according to manufacturers’ instructions and the successful incorporation of mutations was confirmed by DNA sequencing. The plasmid DNAs were transfected into HEK293 cells via a calcium phosphate-DNA co-precipitation protocol. When expressing α1β GlyRs, we transfected the following DNA quantities into each 35 mm culture dish of HEK293 cells: 0.03 μg α1 GlyR, 1.5 μg β GlyR, 0.2 μg neuroligin 2A, 0.2 μg gephyrin, 0.1 μg EGFP. When expressing α3β GlyRs, we transfected the following DNA quantities into each 35 mm culture dish: 0.15 μg α3L GlyR, 1.5 μg β GlyR, 0.2 μg neuroligin 2A, 0.2 μg gephyrin, 0.1 μg EGFP. We have previously validated that these transfection conditions result in a high level of expression of heteromeric α1β and α3β GlyRs, respectively (Zhang et al., 2015).

### Artificial Synapse Formation

Details of this method have recently been published (Dixon et al., 2015; Zhang et al., 2015). Briefly, E15 timed-pregnant rats were euthanized via CO2 inhalation in accordance with procedures approved by the University of Queensland Animal Ethics Committee (approval number: QBI/203/13/ARC). Embryos were surgically removed and placed into ice cold Ca^2+^-Mg^2+^-free Hank’s Balanced Salt Solution under sterile conditions. The spinal cords were then dissected out and pinned at the wider proximal end while meninges were carefully detached. The dissected neurons were then triturated, centrifuged and resuspended in Dulbecco’s Modified Eagles Medium supplemented with 10% fetal bovine serum. Between 40,000 and 80,000 neurons were plated onto each 12 mm poly-D-lysine-coated coverslip in 4-well plates. Neuronal cultures were always maintained in a 5% CO_2_ incubator at 37°C. After incubation for 24 h the entire Dulbecco’s Modified Eagles Medium supplemented plus 10% fetal bovine serum medium was replaced with Neurobasal medium including 2% B27 and 1% GlutaMAX supplements. A second (and final) feed 1 week later replaced half of this medium. Neurons were used in co-culture experiments 1–4 weeks later. Heterosynaptic co-cultures were prepared by directly introducing transfected HEK293 cells onto the primary neuronal cultures 1-3 days prior to electrophysiological recording.

### Electrophysiology

All experiments were performed at 20–22°C at a holding potential of -60 mV. Whole-cell recordings were obtained with a HEKA EPC10 amplifier (HEKA Electronics, Lambrecht, Gremany) and Patchmaster software (HEKA), with currents filtered at 4 kHz and sampled at 10 kHz. Series resistance was compensated to 60% and monitored throughout the recording. Patch pipettes (4–8 MΩ) made from thick-walled borosilicate glass (GC150F-7.5, Harvard Apparatus) were filled with an internal solution comprising (in mM): 145 CsCl, 2 CaCl_2_, 2 MgCl_2_, 10 HEPES, 10 EGTA and 2 MgATP, pH 7.4 with NaOH. Cells were perfused with extracellular solution comprising (in mM): 140 NaCl, 5 KCl, 2 CaCl_2_, 1 MgCl_2_, 10 HEPES, and 10 D-glucose, pH 7.4 with NaOH.

For outside-out recordings, pipettes were fired-polished to a resistance of ∼10 MΩ and filled with the same internal solution. Macroscopic currents in outside-out patches pulled from transfected HEK293 cells were activated by brief (<1 ms) exposure to agonists using a piezoelectric translator (Siskiyou). Currents were recorded using a Multiclamp 700B amplifer and pClamp10 software (Molecular Devices), filtered at 4 kHz and sampled at 10 kHz.

In accordance with standard practice in the field, solutions containing buffered concentrations of free Zn^2+^ were made by adding 10, 100, 500, and 1000 μM free Zn^2+^ to the standard extracellular solution + 10 mM tricine (Sigma-Aldrich) to produce buffered Zn^2+^ concentrations of 0.1, 1, 5, and 10 μM, respectively (Paoletti et al., 1997; Low et al., 2000; Miller et al., 2005b; Vergnano et al., 2014). The added Zn^2+^ concentration was aliquoted from a 10 mM aqueous ZnCl_2_ stock solution on the day of the experiment. ZX1 (Strem Chemicals) was dissolved as a 100 mM stock in water and added to the control solution on the day of the experiment.

### Data Analysis

Analyses of IPSC amplitudes, 10-90% rise times and decay time constants were performed using Axograph X (Axograph Scientific). Single peak IPSCs with amplitudes of at least three times the background noise were detected using a semi-automated sliding template. Events were visually inspected and only well-separated IPSCs with no inflections in the rising or decay phases were included. Current decay phases were fitted with double-exponential functions and a weighted time constant was calculated from individual time constants (τ1, τ2) and their relative amplitudes (A1,A2) as follows: τ_weighted_= (τ1 × A1+τ2 × A2)/(A1+A2). Statistical comparisons were performed using non-parametric tests (Prism 6, GraphPad Software, Inc.). For multiple comparisons we used either the Friedman test or the Wilcoxon matched-pairs signed rank test and for single comparisons we used the Mann–Whitney *U*-test. In all tests we took *p* < 0.05 to be statistically significant. All displayed error bars represent SEM.

## Results

### Zn^2+^-Sensitivity of IPSCs Mediated by α1β, α1^H107N^β, and α1^W170S^β GlyRs

The α1β GlyR is the main synaptic isoform (Lynch, 2009). Zn^2+^ potentiates EC_50_ glycine-gated currents in recombinant α1β GlyRs with an EC_50_ near 40 nM (Miller et al., 2005b) and inhibits with an IC_50_ near 13 μM (Miller et al., 2005a). We also investigated the α1^H107N^β and the α1^W170S^β GlyRs. The H107N mutation eliminates Zn^2+^ inhibition and increases the Zn^2+^ potentiation EC_50_ to 0.8 μM (Miller et al., 2005b). We reasoned that this receptor should respond only to high nanomolar or micromolar concentrations of Zn^2+^. The W170S mutation, which occurs naturally in a sporadic case of human hyperekplexia (Al-Futaisi et al., 2012), eliminates Zn^2+^ potentiation but leaves Zn^2+^ inhibition intact with an IC_50_ near 5 μM (Zhou et al., 2013). We employed this mutant as both a negative control for the potentiating effect of Zn^2+^ and to determine whether Zn^2+^ inhibition (when isolated from potentiation) is capable of affecting IPSC parameters.

In order to precisely control the extracellular Zn^2+^ concentration, we buffered it with 10 mM tricine as described in Section “Materials and Methods.” We also applied 10 mM tricine with no added Zn^2+^ to buffer the free Zn^2+^ concentration to virtually zero, although as recently demonstrated for NMDARs, this may not remove tonic Zn^2+^ that is bound constitutively to low nanomolar affinity Zn^2+^ binding sites (Anderson et al., 2015). Thus, our starting hypothesis is that 10 mM tricine ablates freely diffusing Zn^2+^ but not constitutively bound Zn^2+^.

**FIGURE 1:**
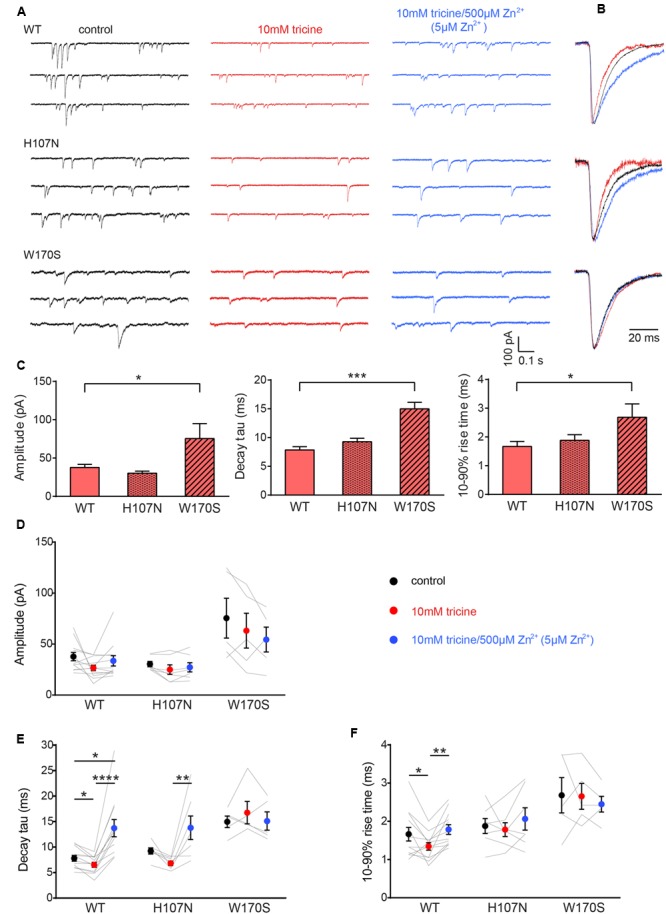
**Effects of synaptic Zn^2+^ on spontaneous IPSCs mediated by α1β, α1^H107N^β, and α1^W170S^β GlyRs. (A)** Representative recordings of IPSCs mediated by the indicated constructs are shown in the presence of tricine-free solution (black traces), 10 mM added tricine (red traces) and 10 mM added tricine + 500 μM added Zn^2+^ (=5 μM free Zn^2+^: blue traces). **(B)** Superimposed, digitally averaged, normalized IPSCs from the corresponding cell in **(A)**. The total number of events included in each averaged IPSC was as follows: α1β GlyR (control: 86; 10 mM added tricine: 63; 10 mM added tricine + 500 μM added Zn^2+^: 92), α1^H107N^β GlyR (control: 72; 10 mM added tricine: 56; 10 mM added tricine + 500 μM added Zn^2+^: 89), α1^W170S^β GlyR (control: 53; 10 mM added tricine: 61; 10 mM added tricine + 500 μM added Zn^2+^: 68). **(C)** Comparison of mean IPSC amplitude, decay time constant and 10–90% rise time for the three constructs in tricine-free solution. Statistical significance was determined by Mann–Whitney *U*-test. The distributions in WT and W170S values differed significantly for amplitude(*U* = 11, ^∗^*p* < 0.05), decay time constant (*U* = 0, ^∗∗∗^*p* < 0.001) and 10–90% rise time (*U* = 10, ^∗^*p* < 0.05). **(D–F)** Comparison of the effects of the indicated solutions on IPSC amplitude, decay time constant and 10–90% rise time for the three constructs. Comparisons between the constructs are not shown. Statistical significance was determined by Friedman test with significance represented by ^∗^*p* < 0.05, ^∗∗^*p* < 0.01, ^∗∗∗^*p* < 0.001, ^∗∗∗∗^*p* < 0.0001. Relative to their values in 10 mM tricine, the decay time constants for α1β and α1^H107N^β GlyRs increased by 74 ± 13% and 50 ± 24%, respectively, in standard extracellular solution and by 109 ± 15% and 101 ± 28%, respectively, in 5 μM free Zn^2+^. We analyzed 12 cells expressing α1β GlyRs, seven cells expressing α1^H107N^β GlyRs and five cells expressing α1^W170S^β GlyRs.

Sample recordings of spontaneous IPSCs from HEK293 cells expressing the indicated constructs are shown in **Figure 1A**. As these IPSCs were almost entirely abolished by 1 μM tetrodotoxin (not shown), we infer they were induced by spontaneous action potentials. The remaining miniature IPSCS were too small to analyze. **Figure 1A** (left) shows sample recordings of spontaneous IPSCs recorded from single cells expressing either the α1β GlyR (top), the α1^H107N^β GlyR (middle) or the α1^W170S^β GlyR (bottom). The left panels show control recordings in tricine-free extracellular solution, the center panels show the effect of 10 mM tricine and the right panels show the effect of adding 5 μM free Zn^2+^. Individual IPSCs from the recordings in A were normalized and digitally averaged to produce the global mean IPSC waveforms as displayed in **Figure 1B**. The mean IPSC amplitudes, decay time constants and 10–90% rise times for each construct recorded in tricine-free extracellular solution are presented in **Figure 1C**. We found that IPSCs mediated by α1^W170S^β GlyRs exhibited significantly larger amplitudes, longer decay time constants and slower rise times relative to those mediated by α1β GlyRs. This indicates that the W170S mutation affects intrinsic receptor gating or synaptic clustering properties in addition to ablating Zn^2+^ potentiation.

**Figures 1D–F** summarizes the effects of adding either 10 mM tricine alone or 5 μM free Zn^2+^ to IPSCs mediated by the three constructs. We observed no effect 10 mM tricine on the IPSC amplitude mediated by any construct (**Figure 1D**). However, the removal of free Zn^2+^ significantly accelerated the decay rate of α1β GlyRs (**Figure 1E**) indicating that the free Zn^2+^ concentration is high enough to positively modulate these receptors. In contrast, 10 mM tricine had no effect on the IPSC decay rate mediated by α1^W170S^β GlyRs. This not only provides a control for the GlyR-specificity of the Zn^2+^ potentiating effect, but also indicates that the low affinity inhibitory effect of Zn^2+^ does not appreciably affect IPSC parameters. All these findings are reinforced by the converse experiment whereby increasing free Zn^2+^ to 5 μM significantly slowed the decay rate in α1β GlyRs and α1^H107N^β GlyRs but had no effect on α1^W170S^β GlyRs (**Figure 1E**). The removal free Zn^2+^ also accelerated the rise times of IPSCs mediated by α1β GlyRs only (**Figure 1F**).

### Zn^2+^-Sensitivity of IPSCs Mediated by α3β GlyRs

**FIGURE 2:**
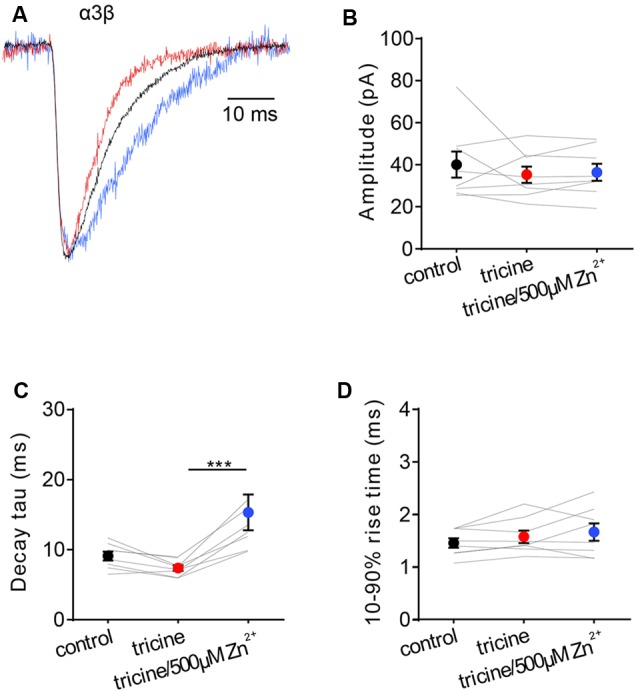
**Effects of synaptic Zn^2+^ on IPSCs mediated by α3β GlyRs. (A)** Superimposed averaged, normalized IPSCs each averaged from >50 events from the same cell. Recordings were made in the presence of tricine-free solution (black trace; averaged from 52 events), 10 mM added tricine (red trace; averaged from 55 events) and 10 mM added tricine + 500 μM added Zn^2+^ (=5 μM free Zn^2+^: blue trace; averaged from 83 events). **(B–D)** Comparison of the effects of the indicated solutions on IPSC amplitude, decay time constant and 10–90% rise time for α3β-mediated IPSCs. All results were averaged from eight cells. Statistical significance was determined by Friedman test with significance represented by ^∗∗∗^*p* < 0.001.

In an attempt to generalize our findings, we investigated the Zn^2+^-sensitivity of the other major adult synaptic GlyR isoform, the α3β, which mediates synaptic inhibition onto nociceptive neurons in superficial laminae of the adult spinal cord dorsal horn (Zeilhofer et al., 2012). Despite their importance in pain signal processing mechanisms and their relevance as novel therapeutic targets for inflammatory pain (Lynch, 2009; Zeilhofer et al., 2012), the Zn^2+^ sensitivity of α3β GlyRs has never been investigated. We thus repeated the experiment of **Figure 1A** on spontaneous IPSCs mediated by α3β GlyRs. Examples of normalized, averaged IPSCs recorded from one cell exposed sequentially to standard extracellular solution containing no tricine, 10 mM tricine, or 10 mM tricine plus 5 μM free Zn^2+^ are shown in **Figure 2A**, with averaged results summarized in **Figures 2B–D**. According to the non-parametric Friedman test, these data reveal that the removal of free Zn^2+^ by 10 mM tricine had no significant effect on any IPSC parameter. However, as all data distributions in **Figure 2B** satisfied the D’Agostino-Pearson omnibus test for normality (using *p* < 0.05 as cutoff), we performed a repeated measures one-way ANOVA on decay time constants of the control versus 10 mM tricine and 10 mM tricine plus 5 μM free Zn^2+^ experimental conditions. As 10 mM tricine significantly accelerated the IPSC decay rate according to this test (*p* < 0.05, *n* = 8), we conclude that the endogenous levels of Zn^2+^ in artificial synapses significantly prolong the decay time constant of α3β GlyR-mediated IPSCs.

### Calibrating the Zn^2+^-Sensitivity of α1β, α1^H107N^β, and α1^W170S^β GlyRs under Simulated Synaptic Activation Conditions

**FIGURE 3:**
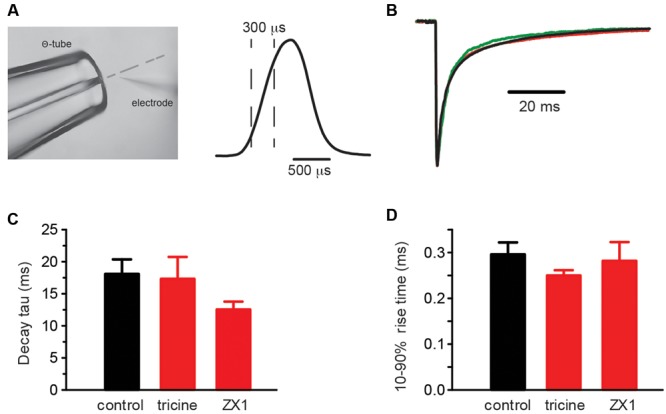
**Control experiments employing rapid solution exchange over macropatches expressing α1β GlyRs. (A,** left) Image of the double-barrelled Θ-tube used to perform the rapid solution exchange experiments on macropatches. The image also shows an open recording pipette and the position of the solution interface (broken line). (*Right*) Open pipette response obtained by switching the solution over the electrode tip from the standard extracellular solution to one diluted to 50% with water. The rise and decay times are approximately 300 μs. **(B)** Overlayed macropatch recordings (averages of 6–20 sweeps) of control (black, 1 mM glycine) and co-application of 1 mM glycine with either 10 mM tricine (red) or 100 μM ZX1 (green). **(C)** Summary bar plots of the weighted decay time constant for control and the two Zn^2+^ chelators. **(D)** Summary bar plots of the rise times for control and the two Zn^2+^ chelators. Mann–Whitney *U*-test revealed no significant differences in the distributions of either the decay or rise times, *p* > 0.05 for both, *n* = 3–8 macropatches for each condition.

The α1β GlyR Zn^2+^ EC_50_ and IC_50_ values cited above were obtained under steady-state applications of Zn^2+^ and EC_50_ glycine (Miller et al., 2005a,b). As these conditions do not apply to the synapse, we calibrated the Zn^2+^ sensitivity of the GlyR constructs using fast solution exchange to mimic synaptic activation conditions. The glycine concentration in the synaptic cleft has been estimated to reach a peak of 1–3 mM in embryonic zebrafish hindbrain neurons (Legendre, 1998) and 2.2–3.5 mM in adult rat spinal motor neurons (Beato, 2008). Glycine is cleared from the cleft with a time constant of 0.6–0.9 ms (Beato, 2008). Considering these parameters, we simulated synaptic activation conditions by applying 1 mM glycine with or without free Zn^2+^ for 0.5–1 ms to outside-out macropatches expressing the GlyR isoform of interest. To calibrate and optimize the solution application system, we rapidly switched the solution perfusing an open patch pipette between standard extracellular solution and an extracellular solution that had been diluted by 50% with distilled water. **Figure 3A** shows an example of an open pipette response, indicating the typical solution exposure profile. We performed this control regularly to ensure that the solution switching rate remained constant within and between experiments.

Contaminating Zn^2+^ released into solution from plastic and glass laboratory ware is thought to reach concentrations as high as 100 nM (Kay, 2004), which is high enough to modulate steady-state GlyR currents activated by low glycine concentrations (Miller et al., 2005b; Cornelison and Mihic, 2014). We determined whether Zn^2+^ contamination might have contributed to the results of **Figures 1** and **2** via two sets of control experiments. The first involved switching rapidly between a control (putatively Zn^2+^-contaminated) solution and a Zn^2+^-chelating solution. In this experiment the control and test solutions both comprised our standard extracellular solution plus 1 mM glycine, although the test solution also contained either 10 mM tricine or 100 μM ZX1 (Perez-Rosello et al., 2015). A sample recording from a single macropatch containing α1β GlyRs suggests the chelators had little effect on decay rate (**Figure 3B**) and the averaged result confirms the lack of a significant effect on either the decay or rise times (**Figures 3C,D**). It is evident, however, that ZX1 showed a tendency to decrease the decay time constant that may become significant with a larger number of observations. If so, this effect could be due to either (1) a direct allosteric inhibitory effect on the GlyR or (2) the removal of tonically bound Zn^2+^ from the GlyR. The second possibility will be investigated below.

In a second control experiment, we initially exposed macropatches to a buffered 100 nM Zn^2+^ solution and switched this rapidly to either a control (putatively Zn^2+^-contaminated) solution (*n* = 10 macropatches) or to a Zn^2+^-chelating solution (i.e., control + 10 mM tricine, *n* = 3 macropatches). Again, no significant differences in rise times or decay rates were observed in either experiment (*p* > 0.05 by Mann–Whitney *U*-test). Together, these results strongly suggest that contaminating Zn^2+^ in our extracellular solution had no significant effect on IPSC rise or decay rates.

**FIGURE 4:**
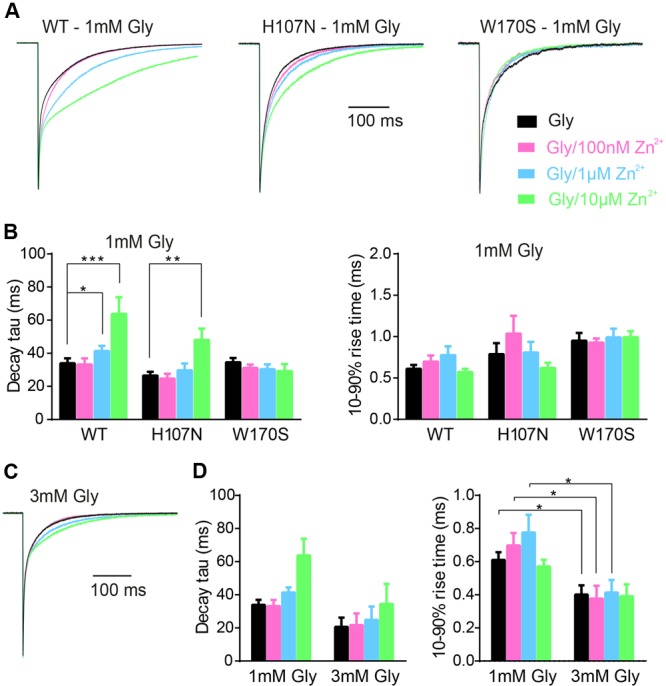
**Calibration of the Zn^2+^-sensitivity of α1β, α1^H107N^β, and α1^W170S^β GlyRs under simulated synaptic activation conditions. (A)** Representative macropatch currents mediated by indicated constructs upon switching from control extracellular solution to one containing 1 mM glycine plus 10 mM tricine plus the indicated concentration of free Zn^2+^. Traces were averaged from >10 sweeps each and normalized. **(B)** Mean values for weighted decay time constants and 10–90% rise times for the three constructs. See Supplementary Table S1 for a full breakdown of n values, mean τ1 and τ2 values and their relative proportions. Statistical significance was determined by Mann–Whitney *U*-test. The distributions in WT (glycine only) and WT (glycine + 1 μM Zn^2+^) values differed significantly for decay time constant (*U* = 63, ^∗^*p* < 0.05, n1 = 17 macropatches, n2 = 13 macropatches). The distributions in WT (glycine only) and WT (glycine + 10 μM Zn^2+^) values also differed significantly for decay time constant (*U* = 20, ^∗∗∗^*p* < 0.001, n1 = 17 macropatches, n2 = 10 macropatches). The distributions in H107N (glycine only) and H107N (glycine + 10 μM Zn^2+^) values also differed significantly for decay time constant (*U* = 16, ^∗∗^*p* < 0.01, n1 = 13 macropatches, n2 = 8 macropatches). **(C)** Representative macropatch currents mediated by α1β GlyRs upon switching from control extracellular solution to one containing 3 mM glycine plus 10 mM tricine plus the indicated free Zn^2+^ concentration. Traces were averaged from >10 sweeps each and normalized. **(D)** Mean values for weighted decay time constant and 10–90% rise time for two glycine concentrations. Statistical significance was determined by Mann–Whitney *U*-test. The distributions in the 1 and 3 mM glycine 10–90% rise time values differed significantly for Zn^2+^ concentrations of 0, 100 nM and 1 μM (*U* = 9–15, ^∗^*p* < 0.05, n1 = 10–17 macropatches, n2 = 5 macropatches).

To mimic synaptic activation, we employed standard extracellular solution without added tricine as the control solution to replicate the conditions employed in **Figures 1** and **2**. Test solutions had the same composition except that we added 1 mM glycine either alone or together with 10 mM tricine and 100 nM, 1 or 10 μM free Zn^2+^. Examples of current responses to the sequential application of each of these four solutions to α1β, α1^H107N^β, and α1^W170S^β GlyRs are shown in **Figure 4A**, with averaged weighted deactivation time constants shown in **Figure 4B**. The averaged individual time constants (τ1, τ2) and their relative amplitudes (A1, A2) for each experimental condition are presented in Supplementary Table S1. For the α1β GlyR, we observed no change in the deactivation rate or 10–90% rise time at 100 nM Zn^2+^. We observed a statistically significant slowing in the deactivation rate at 1 μM and a dramatic slowing in deactivation rate at 10 μM free Zn^2+^. We also observed a statistically significant slowing in the deactivation rate at 10 μM free Zn^2+^at the α1^H107N^β GlyR. At 10 μM Zn^2+^, the percentage increase in the macropatch current deactivation time constant at both receptors (α1β: 76 ± 41%; α1^H107N^β: 76 ± 19%) was comparable to the magnitude of the IPSC decay time constant increase from 10 mM tricine-containing to standard extracellular solution (α1β: 74 ± 13%, α1^H107N^β: 50 ± 24%). In contrast, at the α1^W170S^β GlyR we observed no change in IPSC magnitude, decay rate or rise time at free Zn^2+^ concentrations up to 10 μM (**Figures 4A–C**). Together, these results suggest that the free synaptic Zn^2+^ concentration reaches a concentration of at least 1 μM following a single synaptic stimulation. The lack of an inhibitory effect of 10 μM Zn^2+^ on α1^W170S^β GlyRs is most likely due to a combination of the slow onset of Zn^2+^ inhibition and the brief exposure time to Zn^2+^ under synaptic stimulation conditions.

We next considered the possibility that the magnitude of the Zn^2+^ effect may depend on the synaptic glycine concentration. We therefore repeated the experiment of **Figure 4A** at α1β GlyRs using 3 mM glycine, in line with the maximum predicted synaptic glycine concentration (Legendre, 1998; Beato, 2008). Sample recordings reveal that 10 μM Zn^2+^ exerts a diminished prolonging effect on deactivation rate of currents activated by brief applications of 3 mM glycine (**Figure 4C**). This trend is supported by the averaged data shown in **Figure 4D**. Thus, if the concentration of glycine in the synaptic cleft is 3 mM or higher, our results imply that Zn^2+^ must rise to concentrations greater than 10 μM during synaptic transmission to account for the effect of 10 mM tricine on the IPSC decay rate.

### Effect of a Fast, High-Affinity Zn^2+^ Chelator on IPSC Parameters

**FIGURE 5:**
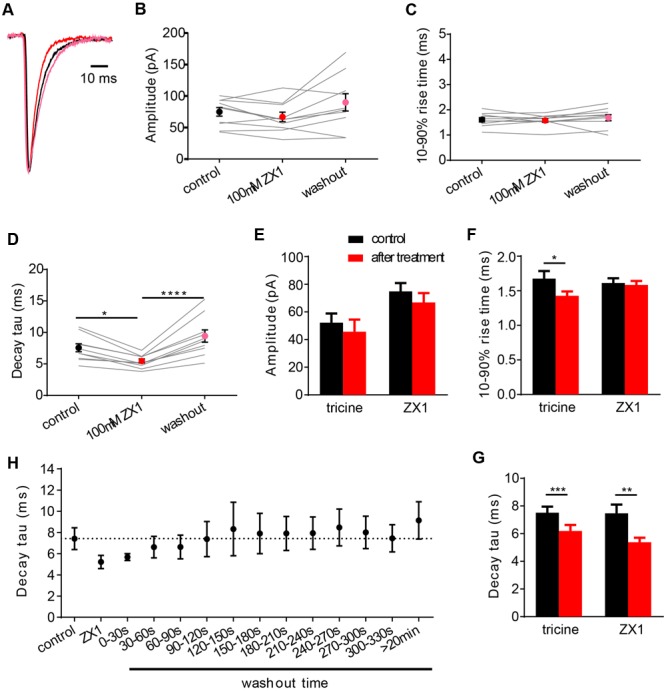
**Effects of 100 μM ZX1 on IPSCs mediated by α1β GlyRs. (A)** Superimposed averaged, normalized IPSCs each averaged from >50 events from the same cell. Recordings were made in the presence of control solution (black trace; averaged from 79 events), after addition of 100 μM ZX1 (red trace; averaged from 61 events) and after 5 min washout (pink trace; averaged from 52 events). **(B–D)** Effects of 100 μM ZX1 on IPSC amplitude, decay time constant and 10–90% rise time for α1β-mediated IPSCs. All results were averaged from 10 cells. Statistical significance in **(D)** was determined by Friedman test with significance represented by ^∗^*p* < 0.05, and ^∗∗∗∗^*p* < 0.0001. **(E–G)** Comparison of the effects of 10 mM tricine and 100 μM ZX1 on IPSC amplitude, decay time constant and 10–90% rise time for α1β-mediated IPSCs. There was no significant difference in the decay time constants in either condition. Statistical significance in **(G)** was determined by two-tailed Wilcoxon matched-pairs signed rank test, with significance represented by ^∗∗^*p* < 0.01 and ^∗∗∗^*p* < 0.001. **(H)** Time course of washout of the ZX1 effect on decay time constant. ZX1 was applied for 3 min. All data points were averaged from five cells.

ZX1 is a fast, high affinity Zn^2+^ chelator (Radford and Lippard, 2013). When applied at a concentration of 100 μM, it has been shown to efficiently remove tonic Zn^2+^ bound constitutively to NMDARs (Anderson et al., 2015). Given that GlyRs and NMDARs both have high affinity Zn^2+^ sites with similar nanomolar affinities, it is reasonable to hypothesize that ZX1 might act similarly on GlyRs. Indeed, a recent study suggested that ZX1 removes tonic Zn^2+^ bound to synaptic GlyRs (Perez-Rosello et al., 2015). We investigated the effects of 100 μM ZX1 on IPSCs mediated by α1β GlyRs both as a control for possible non-specific (e.g., pharmacological) effects of tricine and to investigate the possibility that tonic Zn^2+^ might be bound constitutively to synaptic GlyRs. **Figure 5A** shows examples of normalized, averaged IPSCs recorded from one cell before, during and after exposure to 100 μM ZX1. Results averaged from nine cells recorded under identical conditions reveal that 100 μM ZX1 had no significant effect on IPSC amplitude (**Figure 5B**) or 10–90% rise time (**Figure 5C**) although it significantly reduced the IPSC decay time constant (**Figure 5D**). However, there was no significant difference in the magnitude of the effect of 10 mM tricine and 100 μM ZX1 on the IPSC decay time constant (**Figures 5E–G**). Moreover, in five cells where we quantitated the effects of 10 mM tricine and 100 μM ZX1 sequentially in the same cell, we found the ZX1 exerted no significant additional effect on the IPSC decay time constant (not shown). Thus, if tonic Zn^2+^ is bound constitutively to synaptic α1β GlyRs in our artificial synapses, then its removal has no functional consequence. We thus infer that the effect of tricine and ZX1 on glycinergic IPSCs is mediated by freely diffusing Zn^2+^.

In a final experiment, we quantitated the recovery time course of IPSC decay time constant following ZX1 washout (**Figure 5H**). In this experiment we digitally averaged all IPSCs that occurred within each 30 s interval and fitted a single decay time constant to the averaged waveform. Results were averaged from five cells. As seen in **Figure 5H**, complete recovery from ZX1 application required around 1 min, although the decay time constant eventually stabilized at a value significantly higher than the initial control IPSC decay time constant (**Figures 5D,H**). The recovery from ZX1 inhibition suggests that the concentration of free synaptic Zn^2+^ is not depleted over time under our experimental conditions.

## Discussion

The main advantage of our artificial synapse system is that it allows control over the GlyR subunit composition. However, a potential limitation is that artificial synapses may not faithfully replicate real synapses, especially in terms of synaptic cleft geometry or the location and density of Zn^2+^ and glycine transporters. Although, such factors could affect Zn^2+^ and glycine concentrations in the synaptic cleft, we think they are unlikely to seriously compromise our conclusions for several reasons. First, IPSCs from artificial synapses containing α1β and α2β GlyRs exhibit decay times that are remarkably similar to those from native neuronal synapses expressing the same isoforms (Zhang et al., 2015). Second, the decay time constants of IPSCs recorded *in vitro* from homozygous α1^D80A^ knock-in mice (where Zn^2+^ potentiation is abolished) or from homozygous *Spasmodic* α1^A52S^ mice (where glycine sensitivity is reduced 10-fold) were quantitatively similar to those recorded from artificial synapses incorporating the same mutant subunits (Graham et al., 2006; Hirzel et al., 2006; Zhang et al., 2015). These results suggest a reasonably close correspondence in the concentration profiles of Zn^2+^ and glycine between artificial and real glycinergic synapses. Turning to the present study, tricine-mediated buffering of extracellular Zn^2+^ significantly accelerated the decay rate of IPSCs mediated by α1β and α1^H107N^β GlyRs. However, no change in IPSC amplitude was observed. Together these results imply that the Zn^2+^ concentration in the synaptic cleft is high enough to modulate IPSCs, and that the glycine concentration is saturating. These observations fit well with previous studies on neuronal glycinergic synapses. For example, a study that investigated glycinergic IPSCs in zebrafish neurons found that 10 mM tricine reduced IPSC duration to a similar extent as observed here (Suwa et al., 2001). In addition, studies by Legendre (1998) and Beato (2008) found that the synaptic glycine concentration reaches >1 mM, which is saturating at α1β GlyRs.

By comparing the effects of 10 mM tricine on IPSCs to the effects of co-applied glycine plus free Zn^2+^ to macropatches, we infer that the free synaptic Zn^2+^ rises to at least 1 μM following a single action potential. The dramatic slowing in the IPSC decay rate that we observed in the presence of 5 μM free added Zn^2+^ (**Figure 1E**) is probably due to the total (experimentally applied plus synaptically released) free Zn^2+^ rising to ≥6 μM. In addition, the absence of any effect of Zn^2+^ at concentrations up to 10 μM in the α1^W170S^β GlyR suggests that the well-characterized inhibitory effect of Zn^2+^ (Laube et al., 1995; Nevin et al., 2003; Miller et al., 2005a) is not physiologically relevant, at least at low synaptic stimulation rates. Finally, given the similarity in the effects of 10 mM tricine and 100 μM ZX1 on the IPSC decay rate (**Figure 5G**), we find no evidence for tonic Zn^2+^ binding constitutively to high affinity sites on the GlyR (Perez-Rosello et al., 2015).

It is yet to be demonstrated that Zn^2+^ is co-released with glycine in a phasic manner. However, in the absence of a phasic release mechanism it is difficult to explain how a ≥1 μM Zn^2+^ concentration could be maintained in the cleft for periods typically exceeding 20 min (**Figure 5H**) when the bulk extracellular (tonic) concentration is presumably in the low nM range (Frederickson et al., 2006).

A similar type of analysis of synaptic Zn^2+^ concentration was recently performed on glutamatergic synapses (Vergnano et al., 2014). GluN2A-containing NMDARs exhibit both high affinity Zn^2+^ inhibition in the 10–20 nM range and low affinity inhibition in the 10–50 μM range (Paoletti et al., 1997). Vergnano et al. (2014) compared the effect of 10 mM tricine on GluN2A-mediated synaptic currents from control mice to those from knock-in mice that overexpressed a mutant GluN2A deficient in high affinity Zn^2+^ binding. They found no effect of tricine on single action potential-mediated excitatory post-synaptic currents (EPSCs) in either case, although fast repetitive stimulation unveiled Zn^2+^-mediated inhibition of EPSCs in control animals only. From this they concluded that synaptic Zn^2+^ does not rise high enough to bind to the low affinity sites. It is noteworthy that α1β GlyRs and GluN2A-containing NMDARs have similar high affinities to Zn^2+^. Given that a single action potential releases sufficient Zn^2+^ to modulate glycinergic IPSCs but not NMDAR-mediated EPSCs, we infer that Zn^2+^ rises to significantly higher concentrations in the glycinergic synaptic cleft. Indeed, high affinity chelation of Zn^2+^ by 100 μM ZX1 to remove tonic Zn^2+^ bound to NMDARs is needed to detect an effect of Zn^2+^ released by a single action potential (Anderson et al., 2015). This implies that the Zn^2+^ concentration increase in the glutamatergic synaptic cleft response to a single action potential is in the low nM range.

GlyRs are also potentiated by La^3+^, Pb^2+^, and Co^2+^ and inhibited by Cu^2+^ and Ni^2+^ (Lynch, 2004). As tricine is a low affinity chelator with poor specificity among heavy metals (Ferreira et al., 2015), it is possible that its effect of GlyRs may be due to the chelation of other metal contaminants in the recording solution. Since tricine inhibits glycinergic currents, we infer that any metal contaminant must be a GlyR potentiator. However, the three known potentiators (La^3+^, Pb^2+^, and Co^2+^) are unlikely to be present as contaminants in our recording solutions at concentrations high enough (100 μM) to positively modulate GlyRs. Thus, it is highly likely that the effects of tricine on α1β GlyR-mediated IPSCs is mediated by Zn^2+^. The selectively profile for ZX1 among heavy metals is not known.

Human hyperekplexia, or startle disease, is most commonly caused by hereditary mutations in α1 or β GlyR subunits that disrupt glycinergic neurotransmission (Bode and Lynch, 2014). Most startle mutations result in the complete or partial loss of glycinergic inhibitory function (Chung et al., 2010; Bode et al., 2013; Bode and Lynch, 2014). Given that the human α1^W170S^ startle mutation abolished Zn^2+^ potentiation, it was originally concluded that the startle phenotype was due to a diminished IPSC magnitude or duration (Zhou et al., 2013). In support of this, a startle phenotype had previously been demonstrated in a knock-in mouse overexpressing a mutant α1^D80A^ GlyR subunit in which Zn^2+^ potentiation had been eliminated (Hirzel et al., 2006). Our fast application experiments demonstrate that the inherent deactivation rates of α1β and α1^W170S^β GlyRs in the absence of Zn^2+^ are similar (**Figure 4B**), implying that the loss of Zn^2+^ potentiation in α1^W170S^β GlyRs may indeed lead to a net diminished current carrying capacity. However, in artificial synapses we found that both the mean amplitude and decay rate of IPSCs mediated by α1^W170S^β GlyRs were comparable to those of α1β GlyRs that had been maximally positively modulated by Zn^2+^ (**Figures 1C,E**). This effect could either be due to altered gating properties or differential clustering at the synapse. We have recently shown that the α1^W170S^β GlyR also exhibits spontaneous activation (Zhang et al., 2016) and suggested that, like other gain-of-function startle disease mutations, W170S may cause startle disease via a developmental defect that prevents the maturation of α1β GlyR synapses (Zhang et al., 2016).

## Conclusion

We infer that the free Zn^2+^ concentration in the artificial glycinergic synaptic cleft reaches at least 1 μM following a single presynaptic stimulation. As this concentration is at least an order of magnitude higher than the tonic Zn^2+^ concentration, we assume that diffusible Zn^2+^ is somehow concentrated in the synaptic cleft. The most likely explanation is that Zn^2+^ is phasically released from presynaptic terminals. It is noteworthy that this peak concentration is significantly higher than that reached under similar conditions in the glutamatergic synaptic cleft. We also found no evidence for tonic Zn^2+^ binding constitutively to high affinity GlyR sites. Similarly, we found no evidence for Zn^2+^ inhibition in response to a single action potential in synapses where GlyR Zn^2+^ potentiation had been eliminated. These results provide new insights into the physiological modulatory mechanisms of glycinergic IPSCs.

## Author Contributions

JL conceived the project; YZ and AK performed experiments and analyzed the data; and YZ, AK, and JL interpreted the data and wrote the manuscript.

## Conflict of Interest Statement

The authors declare that the research was conducted in the absence of any commercial or financial relationships that could be construed as a potential conflict of interest.
