# Supplementary material for: The Free Zinc Concentration in the Synaptic Cleft of Artificial Glycinergic Synapses Rises to At least 1 μM
Source: Front Mol Neurosci. 2016 Sep 22;9:88. doi: 10.3389/fnmol.2016.00088 (PMC5031599; doi:10.3389/fnmol.2016.00088)
Supplement: Supplementary file 1 [file Table_1.DOCX]

**Supplementary Information Table 1.** Summary of the fast and slow deactivation time constants, and their relative amplitudes, measured in response to the rapid application of 1 mM glycine to macropatches expressing the indicated GlyR isoforms.

| **GlyR** | **parameter** | **1 mM Gly** | **+100 nM Zn^2+^** | **+1 μM Zn^2+^** | **+10 μM Zn^2+^** |
| --- | --- | --- | --- | --- | --- |
| α1β | τ1 (ms) | 12.0 ± 1.6 (17) | 11.6 ± 1.4 (13) | 12.6 ± 1.4 (13) | 11.0 ± 1.2 (10) |
|  | A1 (%) | 69.0 ± 3.2 (17) | 67.6 ± 3.6 (13) | 61.6 ± 3.1 (13) | 58.0 ± 5.5 (10) |
|  | τ2 (ms) | 87.3 ± 8.3 (17) | 80.3 ± 9.5 (13) | 87.8 ±5.2 (13) | 133.8 ± 13.4**(10) |
|  | A2 (%) | 31.0 ± 3.2 (17) | 32.4 ± 3.6 (13) | 38.4 ± 3.1 (13) | 42.0 ± 5.5 (10) |
| α1^H107N^β | τ1 (ms) | 10.1 ± 1.6 (13) | 11.0 ± 2.1 (10) | 10.7 ± 1.2 (10) | 10.1 ± 1.5 (8) |
|  | A1 (%) | 74.4 ± 4.0 (13) | 75.1 ± 5.0 (10) | 70.0 ± 5.5 (10) | 62.5 ± 5.8 (8) |
|  | τ2 (ms) | 67.3 ± 9.2 (13) | 71.4 ± 16.9 (10) | 64.7 ± 6.3 (10) | 94.0 ± 11.3 (8) |
|  | A2 (%) | 25.6 ± 4.0 (13) | 24.9 ± 5.0 (10) | 30.0 ± 5.5 (10) | 37.5 ± 5.8 (8) |
| α1^W170S^β | τ1 (ms) | 14.1 ± 2.0 (14) | 13.7 ± 2.3 (10) | 13.1 ± 1.7 (10) | 12.9 ± 2.0 (9) |
|  | A1 (%) | 61.4 ± 6.0 (14) | 64.8 ± 6.1 (10) | 58.5 ± 5.7 (10) | 68.5 ± 6.7 (9) |
|  | τ2 (ms) | 61.2 ± 5.3 (14) | 62.0 ± 4.9 (10) | 53.0 ± 3.0 (10) | 59.1 ± 7.4 (9) |
|  | A2 (%) | 38.6 ± 6.0 (14) | 35.2 ± 6.1 (10) | 41.5 ± 5.7 (10) | 31.2 ± 6.7 (9) |
|  | | | | | |

Parameters: τ1 – fast decay time constant; A1 – relative amplitude of τ1; τ2 – slow decay time constant; A2 – relative amplitude of τ2.

n values for each experiment are given in brackets.

** P < 0.01 by Mann-Whitney U test.
